# The Effect of Motivation on Movement: A Study of Bradykinesia in Parkinson’s Disease

**DOI:** 10.1371/journal.pone.0047138

**Published:** 2012-10-15

**Authors:** Tamara Shiner, Ben Seymour, Mkael Symmonds, Peter Dayan, Kailash P. Bhatia, Raymond J. Dolan

**Affiliations:** 1 Wellcome Trust Centre for Neuroimaging, University College London, London, United Kingdom; 2 Department of Clinical Neurology, Cambridge University, Cambridge, United Kingdom; 3 Gatsby Computational Neuroscience Unit, University College London, London, United Kingdom; 4 Sobell Department of Motor Neuroscience and Movement Disorders, University College London, London, United Kingdom; University of Chicago, United States of America

## Abstract

**Background:**

Bradykinesia is a cardinal feature of Parkinson’s disease (PD). Despite its disabling impact, the precise cause of this symptom remains elusive. Recent thinking suggests that bradykinesia may be more than simply a manifestation of motor slowness, and may in part reflect a specific deficit in the operation of motivational vigour in the striatum. In this paper we test the hypothesis that movement time in PD can be modulated by the specific nature of the motivational salience of possible action-outcomes.

**Methodology/Principal Findings:**

We developed a novel movement time paradigm involving winnable rewards and avoidable painful electrical stimuli. The faster the subjects performed an action the more likely they were to win money (in appetitive blocks) or to avoid a painful shock (in aversive blocks). We compared PD patients when OFF dopaminergic medication with controls. Our key finding is that PD patients OFF dopaminergic medication move faster to avoid aversive outcomes (painful electric shocks) than to reap rewarding outcomes (winning money) and, unlike controls, do not speed up in the current trial having failed to win money in the previous one. We also demonstrate that sensitivity to distracting stimuli is valence specific.

**Conclusions/Significance:**

We suggest this pattern of results can be explained in terms of low dopamine levels in the Parkinsonian state leading to an insensitivity to appetitive outcomes, and thus an inability to modulate movement speed in the face of rewards. By comparison, sensitivity to aversive stimuli is relatively spared. Our findings point to a rarely described property of bradykinesia in PD, namely its selective regulation by everyday outcomes.

## Introduction

Bradykinesia is a cardinal feature of Parkinson’s disease (PD) [1] however its precise cause remains the subject of debate, with several hypotheses being put forward [2]–[5]. A principal conjecture is that bradykinesia may be a compensatory response whereby patients slow down in order to improve movement accuracy [2], [3]. This explanation, however, cannot provide a complete picture, as bradykinesia still persists when the spatial accuracy constraints of the task are removed [4], [6]. Another hypothesis proposes that bradykinesia may be due to a deficit in force production [5] this too has contested by studies which have demonstrated that PD are able to achieve adequate muscle contractions on neurophysiological testing [7]. Recent empirical findings and theoretical accounts suggest that bradykinesia, rather than being simply a manifestation of motor slowness (movement speed and initiation), might reflect a specific deficit in the operation of motivational vigour in the striatum. For example, compared with controls, PD patients could achieve similar speeds and accuracy of reaching movements, but did so more rarely, putatively demonstrating an implicit ‘reluctance’ to move fast [8].

A speeding effect of dopamine on action in response to rewards has been widely described [9]–[11]. However, the effect of dopamine depletion on punishment avoidance is much less well understood and has not been formally tested in humans. One of the striking clinical characteristics of bradykinesia in PD is its variability [6], [12], with the same patient being able to achieve very different movement speeds in different contexts. An extreme manifestation of this variability is *“kinesia paradoxica”* where patients are suddenly able to move at near normal speeds, which usually occurs only in extreme aversive contexts [13], [14]. This class of observation motivated us to examine if winnable rewards and avoidable punishments might have differential effects on movement time.

Our use of rewards and punishments furnished us with an opportunity to test whether there is an effect of dopamine depletion, as manifest in the Parkinsonian state, on an ability to maintain a response plan or working memory trace in the face of distraction and whether this is valence specific. This in principle could explain some of the conflicting findings in the literature: PD patients are impaired when required to ‘multitask’ motor and cognitive tasks [15]–[17], although when working memory is explicitly tested, dopamine depletion reduces distractibility [18], [19]. However in these tasks, outcome valence was not explicitly manipulated, leaving unresolved the question of whether an impact of distraction may be context (valence) sensitive.

We developed a novel movement time paradigm involving winnable rewards and avoidable electric shocks, and tested PD patients and matched controls. Critically, we assessed movement time and not reaction times. The motivation here was to remove any confound of cueing, given the known sensitivity of PD patients to visual and auditory cues [20], [21]. Additionally, we were specifically interested in measuring the time it takes to *execute* as opposed to *initiate* a movement, thereby focusing on an important component of bradykinesia. In our paradigm, the faster the subjects performed an action the more likely they were to win money (in appetitive blocks) or to avoid an electric shock (in aversive blocks). We compared patients when OFF dopaminergic medication with controls. This means we tested patients in a more natural disease state, minimising as far as possible the effect of medication fluctuations and dose variations.

## Methods

### Participants

Twenty three adults (12 PD patients and 11 control subjects) participated, with procedures approved by the Moorfields & Whittington Research Ethics committee. Patients were recruited from the National Hospital for Neurology and Neurosurgery (NHNN) and control subjects either through advertisements or in some instances were spouses of patients. Written informed consent was obtained from all subjects and transport costs were reimbursed. Participants were paid an extra fee of between £5 and £15 dependent on task performance.

### Patients with PD

Twelve English speaking early- to moderate-stage [H+Y stage- mean (SE) 2.4 (0.14)] [22] idiopathic PD patients (eight males) (as per UK Brain bank criteria) [age: 48–82 years; mean (SE) 66.6 (2.6) years, 11 right-handed] completed the study. They had on average (SE) 13.25 (0.66) years of education. Initial diagnosis of PD ranged from 3 to 9 years [mean (SE) 5.45 (0.7) years]. There was no history of other major neurological or psychiatric disease. Patients were on various regimens of anti-Parkinsonian medications; carbidopa/levodopa combinations (n = 11); dopamine receptor agonists alone (n = 1). Total daily dose of carbidopa/levodopa varied from 75/300 mg to 250/1000 mg [mean (SE) 117/468 (19.6/78.7) mg].

### Control Group

Eleven English-speaking controls (six males) [age: 38–73 years; mean (SE) 61.72 (3.1) years, 9 right-handed], in good health with no history of neurological or major psychiatric illness completed the study. They had on average (SE) 14.2 (0.8) years of education. Current medications included anti-hypertensives (n = 4), lipid-regulating drugs (n = 3), antidepressants (n = 1), aspirin (n = 1).

Both groups completed the computerised movement time task detailed below. A subsequent neuropsychological test battery was administered, comprising: (i) Mini Mental State Examination to assess cognitive impairment [23]; (ii) Beck Depression Inventory [24]; and (iii) Impulse control disorder questionnaire (adapted from [25]. Severity of clinical symptoms was assessed in the PD group according to the Hoehn and Yahr [22] five-point rating scale, and the Unified Parkinson’s Disease Rating Scale (UPDRS – all sections) [26]. PD patients completed one test session in the relative ‘OFF’ medication state, following a minimum of 12 hours withdrawal from all dopaminergic medication and omission of slow-release preparations for a minimum of 18 hours. Controls also completed one test session. Average ratings were Hoehn and Yahr 2.4 mean (SE) 2.4 (0.14); UPDRS 48.5 mean (SE) 48.5 (3.6). PD patients and controls were well matched for age (F(1,21) = 1.44, p = 0.242 ), education (years) (F(1,21) = 0.87, p = 0.361) and MMSE (F(1,21) = 0.48, p = 0.495). PD patients had higher BDI and ICD scores however when compared to controls the differences only reached trend level significance (BDI (F(1,21) = 3.39, p = 0.08 and ICD (F(1,21) = 3.05, p = 0.095) (table 1)).

**Table 1:** Neuropsychological data sets.

	Patients (n = 12)	Controls (n = 11)
Age	66.6 (2.6)	61.7 (3.1)
Education (years)	13.2 (0.6)	14.2 (0.8)
MMSE	28.5 (0.3)	28.9 (0.4)
BDI	10.2 (1.5)	6.1 (1.5)
ICD	2.25 (0.7)	0.63 (0.54)

Values represent mean (SE). BDI  =  Beck Depression Inventory; MMSE = Mini Mental State Examination; ICD  =  impulse control disorder questionnaire.

### Movement Time Task

Stimulus presentation and response recordings were conducted using Cogent software (www.vislab.ucl.ac.uk), programmed in Matlab (Natwick, MA). The task was designed to measure movement times in response to stimuli associated with rewarding or punishing outcomes. There were two types of trials: trials in which participants’ aim was to win money and trials in which the aim was to avoid shocks. There is no exact way to equate the magnitude of painful shocks with money, but our choice of 10 pence and 1 shock was based on our previous evidence using the same equipment (albeit with a slightly more intense shock) in an (‘implicit’) instrumental performance context [27]. The task consisted of 6 interleaved blocks of 50 trials, blocks of ‘money’ trials alternated with blocks of ‘shock’ trials. The first block type was randomised between subjects.

**Figure 1:**
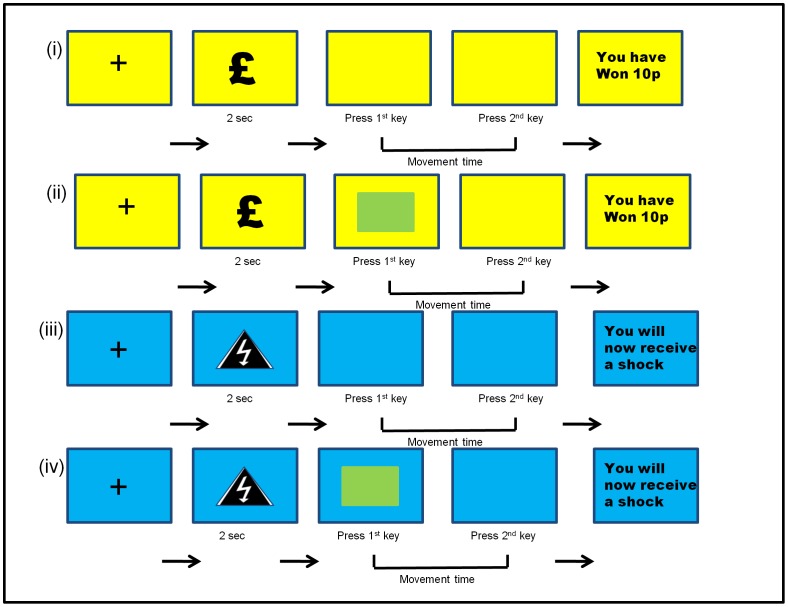
Schematic of the movement time task. Trial types are illustrated as a function of outcome valence (yellow for money trials and blue for shock trials) and presence or absence of a distractor (green flashing square). There were two possible outcomes in the money trials; ‘you have won 10 p’ or ‘you have not won 10 p’ and there were two possible outcomes for the shock trials; ‘you will now receive a shock’ or ‘you will now not receive a shock’. Subjects were exposed to 4 distinct trial types (see methods for details) comprising (i) money trial without distractor, (ii) money trial with distractor, (iii) shock trial without distractor, (iv) shock trial with distractor.

Trials began with presentation of either a money or shock symbol for 2 seconds. Symbols were presented on a blue or yellow background, corresponding to trials in which subjects could win money or trials where they should avoid shocks. This indicator of context was to remind participants of the current trial type. Background colours were counterbalanced across subjects. Participants were instructed to refrain from any action while the symbol remained on the screen. When the symbol disappeared, they were required to press a key on the keyboard to start the trial. Trials were self-paced and only started when the first key was pressed. We opted for this design specifically to prevent the start of the trial being explicitly cued, in light of known effects of cueing in PD [20], [21]. After commencing a trial, by pressing the first key, subjects then needed to press an adjacent key on the keyboard (approximately 1 cm away), using the same finger, in as quick a time as possible. On half of the trials (both in the money and shock trials), after the first key was pressed, an attentional distractor appeared mid screen, which subjects were instructed to ignore. This remained on the screen until the trial was terminated by the second button press (see **Figure 1** for task depiction).

We defined the time between the first and second button press as the movement time. Following the second button press (i.e. completion of the trial), a screen was shown indicating trial outcome. In ’money’ blocks, participants either did or did not win 10 p. In ‘shock’ blocks, participants either avoided or received a shock. Outcome delivery depended probabilistically on the reaction time, such that the probability of receiving a reward (or avoiding a shock) was varied inversely with the reaction time. However the constant of proportionality varied slowly over the course of the experiment to induce additional variability, so that at times a fast reaction was very likely to result in successful reward or avoidance, and at other times less likely. This was done to ensure that subjects remained engaged with the task and did not 'habituate' the task parameters. Specifically, the function used was p (reward or avoidance)  = 1−(c(t) * RT(t) ), where c(t) is a slowly changing Gaussian random walk over trials t and bounded between 0.2 and 1, and RT(t) is the reaction time on trial t.

Subjects first performed a short practice session in order to familiarise themselves with the task during which they neither received shocks nor won money.

Participants were seated in a well-lit room in front of a desktop computer with a normal keyboard. Two Digitimer boxes were fitted with circular electrodes. Triggers for the shock box were sent via the parallel port to the input on the shock box. Before commencing the task, participants had an electrode attached to the back of their non-dominant hand and underwent a shock titration procedure. This consisted of first establishing a maximal threshold level at which the electrical current was rated as very uncomfortable. Then, an automated staircase procedure was used to determine the level of shock for each individual that was 60% of their own maximal threshold.

Failure to complete a trial correctly, for example by pressing the same button twice in error, resulted in no outcome being delivered (i.e. no money or shock outcomes). Overall, there were very few trials where subjects failed to respond [mean (SD), 2.47 (4.35) from a total of 150 trials]. Thus, there was no indication that subjects used this as an ‘escape route’ from aversive outcomes. Failure to respond on one trial did not impact on movement time on subsequent trials and mean movement time before, and after, this contingency was utilised did not differ (ttest p>0.2).

### Data Analysis

We initially focused on the overall effects of disease on movement time, examining the differences in performance in the money versus the shock trials, and comparing the effects of these outcomes with those in the control group. We also examined effects of previous trials’ outcomes on the movement times of subsequent trials by performing multiple regression analysis. Here we modelled separately the modulatory effects of receiving money compared with not receiving money on the previous trial; and the effects of receiving shock compared with not receiving a shock on the previous trial. We also included terms for the overall average effect on movement time of money and shock trials, anticipating that these would be different. Beta values estimated from the regression model were entered into one sample t-tests at the group level to make inferences about the effect size of four factors:
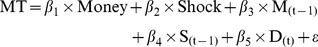



Where:

MT  =  movement time.

Money  =  indicator variable for all money reward trials. β_1_ corresponds to the average MT for money trials.

Shock  =  indicator variable for all shock punishment trials. β_2_ corresponds to the average MT for shock trials.

M_(t−1)_ = indicator (1/−1) of outcome of previous money trial. β3 corresponds to the modulatory effect of receiving money at trial t−1.

S _(t−1)_ = indicator (1/−1) of outcome of previous shock trial. β_4_ corresponds to the modulatory effect of receiving a shock at trial t−1.

D_(t)_ =  indicator (1/−1) of whether distractor present. β_5_ corresponds to the modulatory effect of a distractor at trial t.

ε =  error term.

Terms were entered simultaneously into the regression (i.e. without orthogonalisation). Additionally, we performed an ANOVA examining the effect of the distractor on movement times, testing for a 3-way interaction between block type (money/shock), distractor (present/not present) and group (controls/patients).We also looked at the time taken from the appearance of the money or shock symbol until the first button press. This was to confirm there were no differences in movement time between the groups or valence conditions which could indicate differences in motor preparation times. We excluded data from blocks where movement times in the first block were over 150% longer than the movement times in subsequent blocks for the same type of trial (one money block in a control and one shock block in a patient). We believe this incongruous performance in these subjects reflected an initial failure to understand the task demands which led to performance changing drastically between the first and subsequent blocks.

## Results

**Figure 2:**
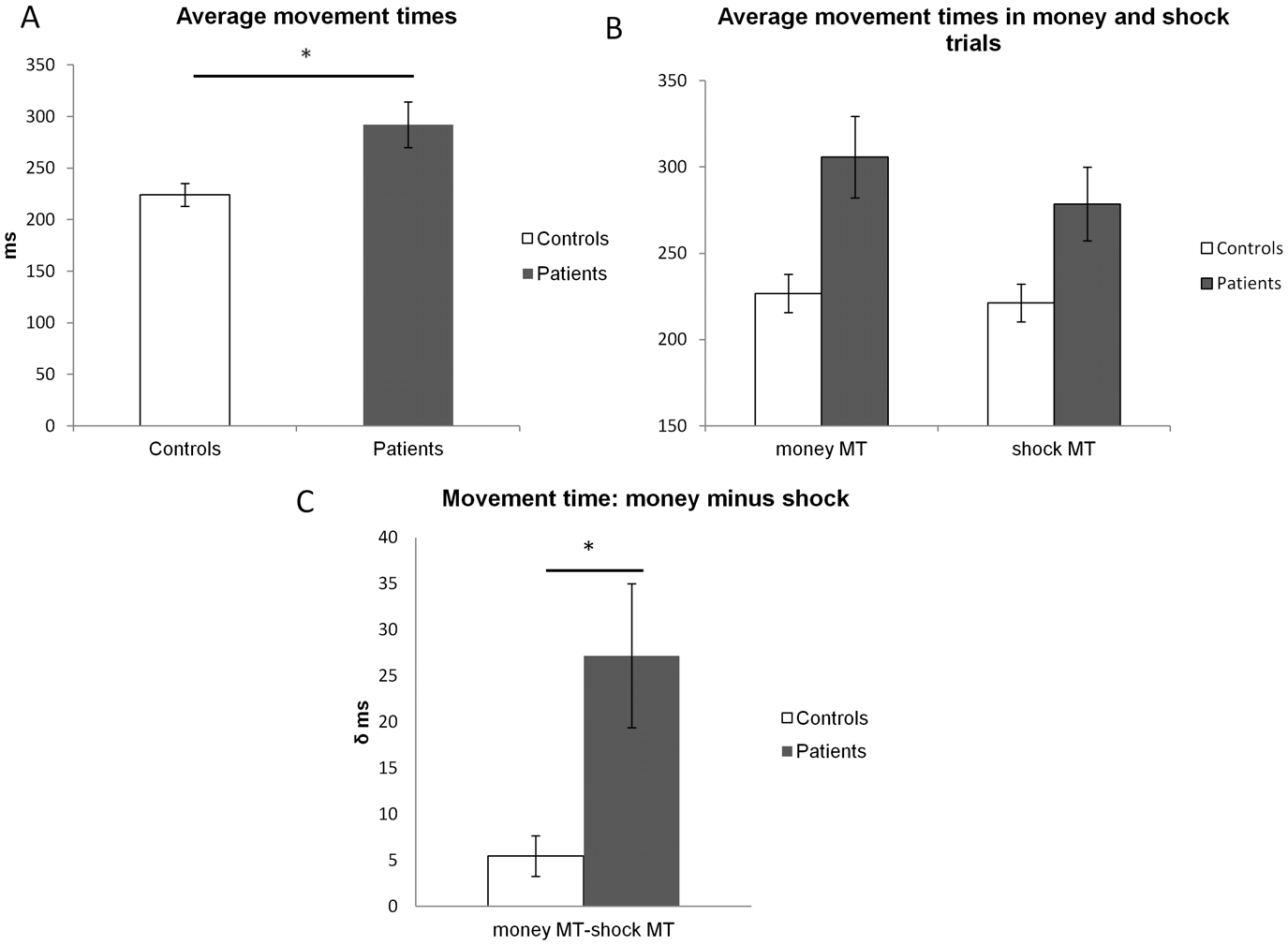
(A) Average movement times. Average movement times (MT) (in ms) collapsed across money and shock trials for controls (white bars) and patients (grey bars). Error bars represent (two times) the s.e.m. (B) **Average movement times in money and shock trials**. Average movement times in money and shock trials for controls (white bars) and patients (grey bars). Error bars represent (two times) the s.e.m. (C) **Money MT minus shock MT.** Differences in movement time in money compared with shock trials. Plotted is the difference in movement times (ms) in money trials minus movement time in shock trials for controls (white bars) and patients (grey bars). Error bars represent (two times) the standard error of the difference.

Our analysis indicated two main effects. First, we found an effect of group whereby patients were slower overall than controls F (1,21) = 15, p = 0.001 (**Figure 2A**). Second, we found an effect of valence such that both patients and controls were faster for shock compared to money trials (paired t-tests comparing money with shock trials in controls T (1,10) = 2.51, p = 0.031 two tailed; and in patients T(1,11) = 3.49, p = 0.005 two tailed) (**Figure 2B**).

Crucially we observed an interaction between group (control/patients) and outcome valence condition (shock/money) F(1,21) = 6.6, p = 0.017. The interaction was characterised by a bigger *difference* in movement time (MT) between money trials and shock trials in patients compared with controls (**Figure 2C**).

We next examined the effect of outcome in a previous trial on movement time in the subsequent trial. We hypothesised that movement times would be influenced both by context (i.e. money compared with shock trials) and also experience on a previous trial, evident in a trial-by-trial sensitivity to rewards and punishments. For example, we expected that failure to achieve the desired outcome (i.e. not winning money or receiving a shock) on a previous trial would lead to faster movement on the subsequent trial. This is what we found for the control group in the case that they failed to win money (T(1,10) = −2.23, p = 0.049 two tailed). However, this speeding effect was absent in patients (T(1,11) = −1.23, p = 0.242 two tailed) indicating that controls were not simply operating at ceiling speed in the money trials as they modulated their movement times based on trial-by-trial outcomes while PD patients did not. Both patients and controls responded in the same manner to receipt of a shock by tending to improve their speeds in the trials following shocks, however, this speeding was not statistically significant.

In addition we found no differences in speed between the early and late trials (t-test comparing average MT in first block compared with last block; in patients for money trials T(1,11) = −0.643, p = 0.533, for shock trials T(1,11) = −1.074, p = 0.306; for controls for money trials T(1,10) = 0.251, p = 0.807, for shock trials T(1,10) = −0.960, p = 0.360) indicating that there was no evidence of learning over the course of the blocks.

To ensure that the faster responding for shock in the patient group could not be explained by a prolonged motor preparation time, we examined the time taken from the symbol appearance to the first button press There was no significant difference in this initial period of time between the groups (patients/controls) F(1,21) = 0 (p = 0.997) or conditions (shock/money) F(1,21) = 0.557 (p = 0.464) or an interaction between group and condition F(1,21) = 2.37 (p = 0.138) ruling out this possibility.

**Figure 3:**
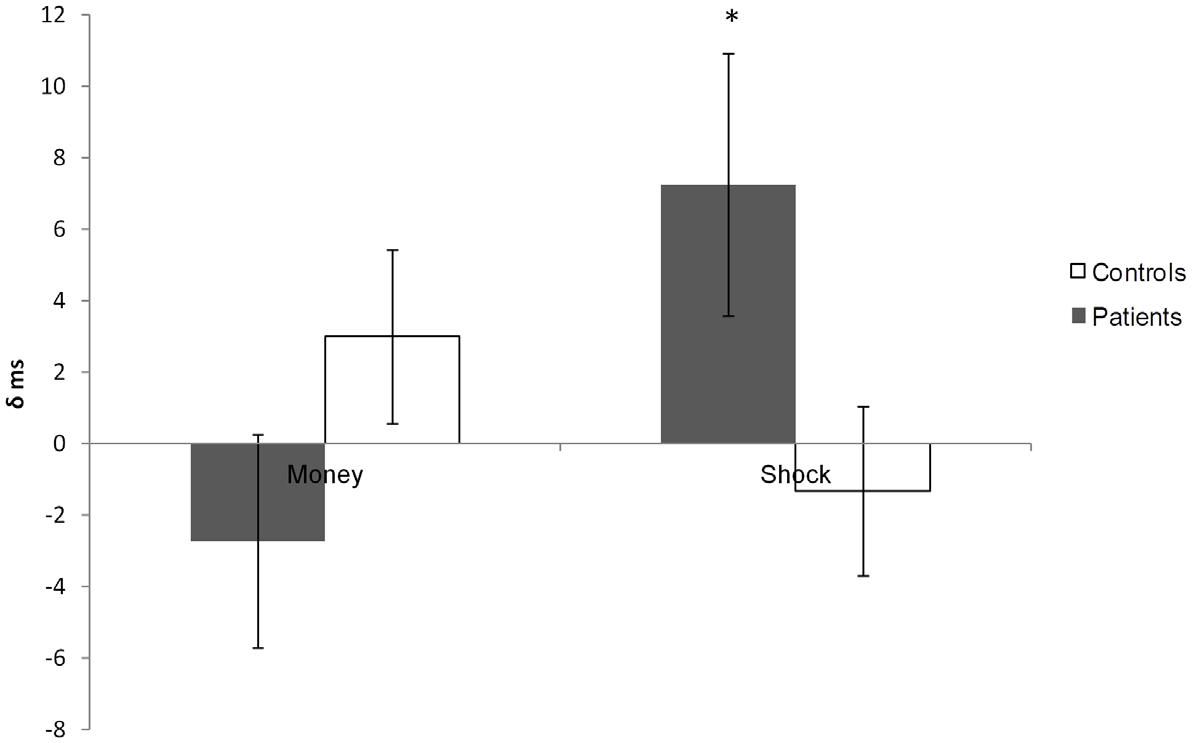
Distractor MT minus no distractor MT. Plotted is the difference in movement time (ms) in trials with a distractor present minus movement time in trials with no distractor present, for money trials and shock trials separately. Controls are represented by white bars and patients by grey bars. Error bars represent (two times) the standard error of the difference.

A differential effect of distractor on movement time was evident. In the repeated measures ANOVA there was a significant 3 way interaction between block (money/shock), distractor (present/not present) and group (controls/patients) (F (1,21) = 7.54, p = 0.012) characterised by patients’ movement time slowing when performing a shock trial where a distractor was present (**Figure 3**).

Our multiple regression analysis allowed us to look for more subtle differences in the modulatory effect of previous trials on movement time in the two groups while controlling for other factors. This confirmed our findings that not winning money in a previous trial had a significant effect on movement time in the control group (one sample t-test p<0.001 two tailed. β_3 (controls: effect of loss at t−1)_: mean (SE) 3.86 (0.7)), showing that controls sped up significantly on a trial after they failed to win money. This speeding effect was absent in patients (one sample p = 0.458, two tailed β_3 (patients: effect of loss at t−1)_: mean (SE) 2.15 (2.7)).

## Discussion

Our most notable result is a valence asymmetry in the movement time of PD patients. This comparative failure to speed up in order to win rewards is consistent with previous findings in PD patients OFF medication [11] and supports proposals that tonic dopamine levels control the rate and vigour of movements, possibly by signalling the average reward rate in the environment [10], [28]. This notion has been linked to the idea of impaired ‘motor motivation’ in PD, whereby there is a shift in the cost/benefit ratio of moving fast [8]. Crucially, we find that although the response to rewards appears impaired in the PD group when compared with controls, the trial-by-trial response to punishments is not similarly impacted, a fact which has not previously been demonstrated. This finding highlights that in PD, dopamine depletion has a lesser impact on responses to punishments compared to rewards, and hints at a more complex role for dopamine in active avoidance. A critical aspect to our task is that we examined the effect of explicit contexts on movement time and compared subjects in dopamine depleted and non-dopamine depleted states. Our findings indicate that bradykinesia is not simply related to movement, but rather to the way in which a hypodopaminergic striatum computes action values.

Importantly, we observed a difference in the effect of past monetary loss on subsequent actions in patients compared with controls, where subjects were given trial-by-trial feedback on whether their performance sufficed to merit a reward or avoid a punishment. If learning is effective, we expect a speeding up of movements following trials with negative outcomes (failure to win money or avoid a shock), thereby improving the chances of achieving the desired outcomes on subsequent trials. This effect was evident in the control group for rewards, but was absent in the PD patients. Patients did not speed up their movements after failing to win a reward despite physically being able to move faster, a fact they clearly demonstrated in the shock avoidance trials. Parkinson’s disease results in deficits across several cognitive domains, including probabilistic learning and classification tasks [29], [30] with dopamine replacement therapy (DRT) having distinct effects on these behaviours. The observation of failure to adjust speed in the face of monetary loss in PD patients is consistent with findings of impaired reward feedback learning in PD patients OFF medication which has been shown in previous studies [31]–[33], often postulated to be due to decreased reward prediction error magnitude in response to positive outcomes. Of note, this trial-by-trial adaptation, whereby subjects speed up in response to a failure to win money has been observed previously albeit in the context of a probabilistic task in which this speeding was evident in both controls and patients [11]. One further possibility would have been to compare trials with valenced outcomes with neutral trials, however this risks lengthening the experiment and reducing power and increasing fatigue, and behaviour towards neutral cues can be difficult to interpret [34].

Finally, we found a detrimental effect of a distractor that was only evident in the shock trials in PD patients, indicating that here too there is an asymmetrical effect of valence. The context specificity of distraction has been demonstrated previously, with susceptibility to distraction being higher in PD patients when multitasking is required [15]–[17] but lower in working memory tasks when OFF medication [18], [19]. Here we show that distraction is also valence specific. PD patients can improve both their motor speed and accuracy of their movements with increased attention [35], [36], and we propose that in our study, the hypodopaminergic state in PD leads to decreased attending to appetitive stimuli compared with aversive stimuli, leading to improved motor performance at the cost of an increased sensitivity to distraction in the aversive trials. This fits neatly with the known reduced sensitivity of patients to rewards [32].

As this was not an imaging study we can only speculate on the neural mechanism underlying the differences in behaviour observed in the PD patients. Previous research has pointed to compensatory increases and/or modulation of cortical activations in PD patients carrying out motor tasks [37], [38]. It is possible that the trials with the negatively valenced outcomes influenced this pattern of cortical engagement possibly by an interplay between the motor and limbic fronto-striatal circuits [14] driving better performance in these trials. In view of the fact that the effect of distraction was significantly greater in the shock trials it may well be that attentional factors play the biggest role in the modulation of this cortical engagement. This would be interesting avenue to pursue in the future with an imaging experiment.

In sum, we provide evidence that bradykinesia is in part a context dependent deficit. We link the cognitive and motor deficits associated with the PD hypodopaminergic state by demonstrating that bradykinetic movements are dependent on the valence frame in which movements are executed. Such modulation is apparent in *“kinesia paradoxica”*, where PD patients can suddenly move quickly in exceptional circumstances [13], [14] eloquently described by Mac-Donald Critchley in 1929 who noted: “The influence of the emotions upon these more automatic movements is frequently striking; under the influence of sudden fear or during an instinctive response to danger, the gait may lose its feeble and short-stepping characters and approximate to the normal.” By using monetary gains and losses and comparing them with physical shocks we aimed to recreate the aversive circumstances under which kinesia paradoxica has been described [13]. Here we showed this effect in a controlled environment with conventional cues whose motivational salience is internally rather than externally assessed. In the future it would be interesting to see whether varying the magnitude of both the positively and negatively valenced outcomes might modulate this behavioural effect. Additionally we demonstrated that distractors play an important role in performance in PD patients and that this effect is also valence specific. A better understanding of the impact of valence on movement may inform the future development of new strategies to increase the effectiveness of rehabilitation treatments.
